# Multiclass Motor Imagery Recognition of Single Joint in Upper Limb Based on NSGA- II OVO TWSVM

**DOI:** 10.1155/2018/6265108

**Published:** 2018-06-28

**Authors:** Shan Guan, Kai Zhao, Fuwang Wang

**Affiliations:** School of Mechanical Engineering, Northeast Electric Power University, 132012 Jilin, China

## Abstract

In the study of the brain computer interface (BCI) system, electroencephalogram (EEG) signals induced by different movements of the same joint are hard to distinguish. This paper proposes a novel scheme that combined amplitude-frequency (AF) information of intrinsic mode function (IMF) with common spatial pattern (CSP), namely, AF-CSP to extract motor imagery (MI) features, and to improve classification performance, the second generation nondominated sorting evolutionary algorithm (NSGA-II) is used to tune hyperparameters for linear and nonlinear kernel one versus one twin support vector machine (OVO TWSVM). This model is compared with least squares support vector machine (LS-SVM), back propagation (BP), extreme learning machine (ELM), particle swarm optimization support vector machine (PSO-SVM), and grid search OVO TWSVM (GS OVO TWSVM) on our dataset; the recognition accuracy increased by 5.92%, 22.44%, 22.65%, 8.69%, and 5.75%. The proposed method has helped to achieve higher accuracy in BCI systems.

## 1. Introduction

BCI is a technology that enables the brain to establish communication and control directly between human brain and computer or other electronic devices without the help of peripheral nerves and limbs [1, 2]. BCI technology not only enhances the ability of disabled patients to communicate with the outside world in the field of medical rehabilitation [3, 4], but also has wide applications in smart home, mass consumption and entertainment, military, and other fields. At present, the research direction of BCI system is mainly in the following aspects: sensorimotor (SMR) [5], slow cortical potential (SCP) [6], P300 event-related potential [7], and steady-state visual evoked potential (SSVEP) [8]. The most widely used is the SMR BCI system based on motor imagery.

The *μ* (8-13Hz) and *β* (13-30Hz) rhythms in EEG signals will cause a phenomenon named event-related desynchronization (ERD) and event-related synchronization (ERS) when motor imagery occurs [9, 10]. This means that the rhythmic activities of the brain represent frequency specific changes may consist either of decreases or of increases of power in given frequency bands. The ERD/ERS phenomenon is an important basis for the BCI systems of motor imagery.

In order to improve the classification accuracy of the BCI system, researchers have studied the feature extraction methods and classification methods of EEG signals [11–15]. The most commonly used feature extraction methods include wavelet packet transform (WT), Fourier transform (FT), CSP [16, 17], and autoregressive (AR) model. The classification methods include linear discriminant analysis (LDA), support vector machine (SVM), neural network (NN), and so on. Wang et al. used the convolution neural network (CNN) to recognize the image of the brain topographic map of three kinds of motor imagery movements of the upper limb, flexion wrist, and wrist external rotation, and the highest recognition rate in the three classification experiments is 67.89% [18]. Roy et al. carried out Hilbert transform for two kinds of motor imagery of shoulder and elbow joint and used discrete wavelet transformation to extract features; SVM gets the highest recognition rate of 84.91% in the five recognition methods [19]. Sachin et al. used empirical mode decomposition (EMD) to extract the energy features of left and right hand motor imagery EEG signals and classified them using LS-SVM; the recognition rate is no less than 95.56% [20]. Tang et al. used PSO to optimize the hidden-layer visible deep stacking network (HVDSN) to recognize the left and right hand motor imagery EEG signals; the recognition rate is no less than 89.84% [21]. Although the above studies have achieved high recognition results, most of the research focuses on hands and feet motor imagery, few studies have been conducted based on multiclass motor imagery of single joint.

In this paper, we propose a method to improve accuracy of motor imagery BCI using AF-CSP and an optimized OVO TWSVM classifier. The proposed method is composed of a total of four stages. First, a notch filter and common average reference (CAR) are used to remove noise in EEG signal. Second, EMD is used to obtain IMF, and FFT is used to obtain AF information of the IMF. Third, the NSGA-II is used to tune hyperparameters for linear and nonlinear OVO TWSVM. Finally, an optimized OVO TWSVM classifier is evaluated using laboratory data sets (three kinds of motor imagery of shoulder flexion, extension, and abduction) and compared with state-of-the-art algorithms (LS-SVM, BP, ELM, PSO-SVM, and GS OVO TWSVM).

## 2. Materials and Methods

**Figure 1:**
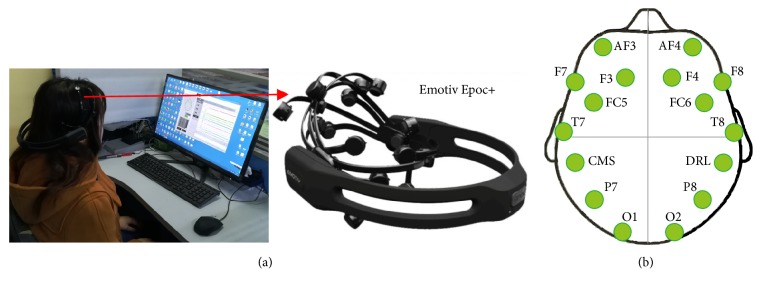
(a) Experimental photos and Emotiv Epoc+ and (b) Emotiv 14 electrodes located over 10-20 international system positions.

**Figure 2:**
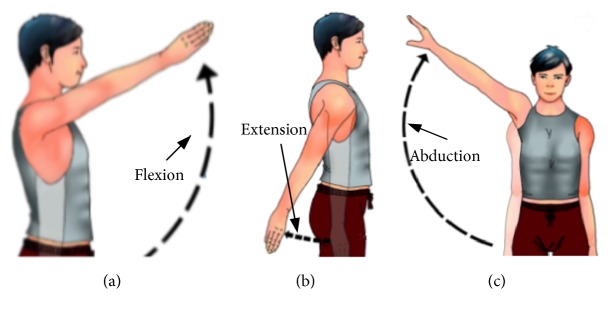
Three movements of shoulder joint: (a) flexion, (b) extension, and (c) abduction.

Emotiv Epoc+ is used to collect EEG data of motor imagery. It is a portable EEG acquisition device with a sampling rate of 128Hz. It has fourteen electrode channels (AF3, F7, F3, FC5, T7, P7, O1, O2, P8, T8, FC6, F4, F8, and AF4) and two inference electrodes (CMS, DRL), and the electrode placement follows the international 10-20 standard. Experimental photos, equipment, and the Emotiv 14 electrodes are located over 10-20 international system positions as shown in Figure 1. This experiment collected three kinds of EEG signals of one joint: imagination of shoulder flexion, extension, and abduction, as shown in Figure 2.

**Figure 3:**
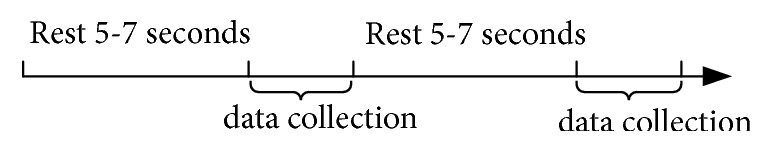
Timing for experimental process.

Seven subjects participated in this experimental study. These subjects were in good health. During the experiment, subjects were naturally placed with both hands, trying to avoid body or head movement. During the experiment, subjects carried out motor imagery under the outside cue, a single experiment collected EEG signal for 5 seconds, and then take 5-7 seconds to have a rest; each action repeated acquisition 20 times. The experimental process is shown in Figure 3. The dimension of EEG is high and the amount of data is large, in order to reduce the computational complexity. In this paper, the EEG signals collected from four electrode channels of FC5, F3, F4, and FC6 were selected for the following motor imagery analysis.

## 3. Theories and Methods

### 3.1. Data Preprocessing

EEG signals contain a variety of noise, and it is necessary to perform spatial filtering before feature extraction of the signal. First, the 50Hz notch filter is used to remove the power frequency noise. McFarland et al. compared four kinds of spatial filtering technology to improve the SNR of EEG signal, and the conclusion shows the superiority of CAR and large Laplacian methods [21]; this paper uses CAR method as the spatial filter. The calculation of CAR is to subtract the average of all the electrodes from the selected channel. The formula for calculation is as follows:

(1) $$\begin{array}{c} V_{i}^{CAR}=V_{i}^{RAW}-\frac{1}{4}\overset{4}{\underset{j=1}{\sum }}V_{j}^{RAW} \end{array}$$

where *V*_*i*_^*CAR*^ is filtered potential and *V*_*i*_^*RAW*^ is the potential of the *i* electrode.

### 3.2. Empirical Mode Decomposition

EMD is used to stabilize the nonstationary signals and obtain IMF. The specific EMD decomposition process is given in document [22]. EEG signal can be decomposed into IMF components after the empirical mode decomposition, and the expression is as follows:

(2) $$\begin{array}{c} S\left(\;t\;\right)=\overset{N}{\underset{i=1}{\sum }}C_{i}\left(\;t\;\right)+R_{n}\left(\;t\;\right) \end{array}$$

where *S*(*t*) represents the original EEG signal, *C*_*i*_(*t*) is the *i*^*th*^ IMF, and *R*_*n*_(*t*) is residual components after screening.

**Figure 4:**
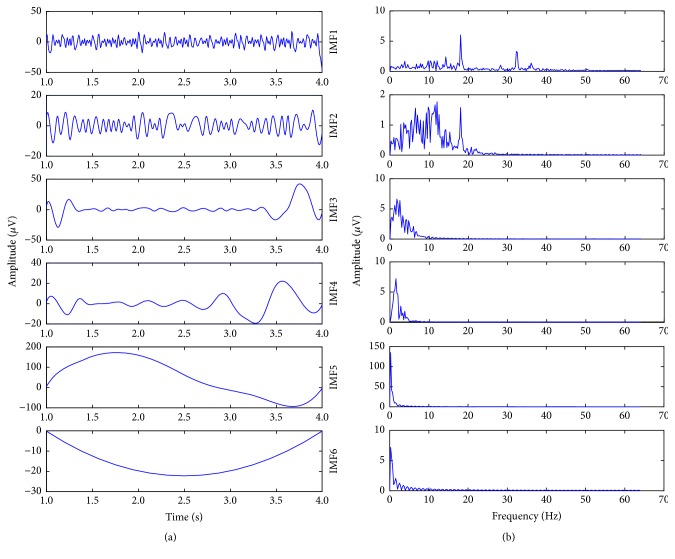
(a) IMF obtained from 3s EEG signals after EMD decomposition. (b) Amplitude-frequency domain information corresponds to per IMF of Figure 4(a).

Taking the 1-4s data of the F3 electrode channel in Figure 3, as an example, use EMD method to decompose the denoised signal, and the IMF component is shown in Figure 4(a).  Figure 4(b) is the AF domain information for the IMF component after FFT. From Figure 4(b), we can see that the *μ* and *β* rhythms of the motor imagery are mainly distributed in IMF1 and IMF2. In this paper, the sampled data of the selected 4 electrode channels are decomposed by EMD, respectively, do FFT to IMF1 and IMF2 of each electrode channel, and construct the *i*^*th*^ experiment EEG data matrix *X*_*i*_ (*i* = *N* × *M*), where *N* corresponds to this article is 8 and *M* is the selected information of *μ* and *β* rhythms.

### 3.3. Common Spatial Pattern

The traditional CSP algorithm is essentially looking for a spatial filter to obtain more obvious eigenvectors after the signal passed through the filter, which makes a kind of signal variance reach a maximum, and another signal reaches a minimum. This method achieves the purpose of distinguishing two types of signals. Combining AF information of IMF with CSP to form AF-CSP and applying one versus one (OVO) strategy to AF-CSP make AF-CSP suitable for multiple classification problems, and the specific process is as follows:

(3) $$\begin{array}{c} R=\frac{X_{i}X_{i}^{T}}{trace\left(X_{i}X_{i}^{T}\right)} \end{array}$$

where *T* is transpose operator. Then calculate the mixed space covariance matrix *R*_*c*_ of the two types motor imagery *R*_*l*_, *R*_*r*_ as follows:

(4) $$\begin{array}{c} R_{C}=R_{l}+R_{r} \end{array}$$

Eigenvalue decomposition for covariance of mixed space is as follows:

(5) $$\begin{array}{c} R_{C}=U_{C}A_{C}U_{C}^{T} \end{array}$$

where *U*_*C*_ is the eigenvector matrix and *A*_*C*_ is the eigenvalue diagonal matrix. Whitening matrix *P* is calculated in

(6) $$\begin{array}{c} P=A_{C}^{-\text{1/2}}U_{C}^{T} \end{array}$$

The whitening matrix causes eigenvalues of transformed matrix be equal one, so we calculate transformed covariance matrixes *S*_*j*_ and *S*_*k*_ in

(7) Sj=PRlPT

(8) Sk=PRrPT

After whitening, the matrixes *S*_*j*_ and *S*_*k*_ have the same eigenvector, and the following formula can be obtained after the eigenvalue decomposition:

(9) Sj=BATBT

(10) Sk=BARBT

The desired spatial filter is obtained by the upper form *W* = (*B*^*T*^*P*)^*T*^, and we can get a new data matrix after filtering by *W*: *Z*_*N*×*M*_ = *W*_*N*×*M*_*X*_*i*_.Feature vectors can be obtained by

(11) $$\begin{array}{c} f_{p}=\log\left(\;\frac{\mathrm{var}\left(Z_{p}\right)}{\sum_{i}^{2m}\mathrm{var}\left(Z_{i}\right)}\;\right) \end{array}$$

where *Z*_*p*_ (*p* = 1,…, 2*m*). The dimension of *f*_*p*_ can not exceed *N* at most; in this paper, we set *m* = 2, so we can get a vector of four dimensions.

Combined with OVO strategy, eigenvectors constructed between every two types of actions are

(12) f1=F1,E1

(13) f2=F2,A1

(14) f3=E2,A2

where *f*_1_ represents the eigenvector obtained after AF-CSP transformation of flexion (*F*_1_) and extension (*E*_1_) of shoulder joint. *f*_2_ represents the eigenvector obtained after AF-CSP transformation of flexion (*F*_2_) and abduction (*A*_1_) of shoulder joint. *f*_3_ represents the eigenvector obtained after AF-CSP transformation of extension (*E*_2_) and abduction (*A*_2_) of shoulder joint.

**Figure 5:**
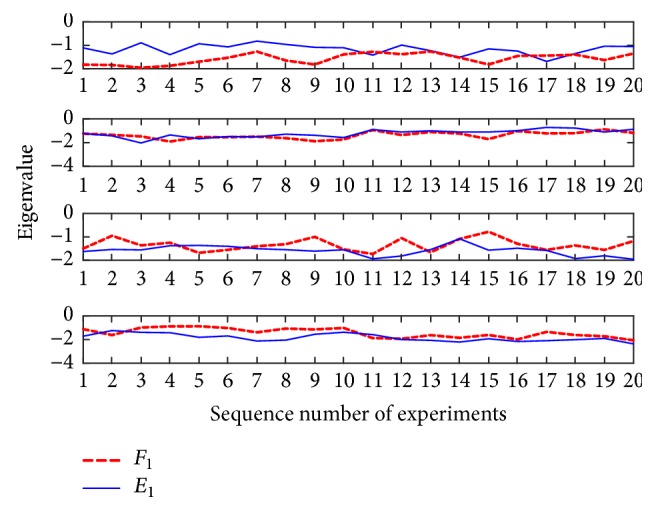
Twenty sets of four-dimensional eigenvectors *f*_1_ obtained by AF-CSP method of flexion and extension of shoulder joint.

Figure 5 shows one example of the eigenvector *f*_1_ constructed by AF-CSP of subject B, and *f*_1_ is constructed by two MI tasks of shoulder joint flexion and extension. In Figure 5, the lateral axis represents the sequence number of experiments and the vertical axis represents the eigenvalue. We can see clearly from Figure 5 that the selected 4-dimensional feature vectors constructed by AF-CSP are distinctly distinguishable. The final eigenvector constructed by the AF-CSP method is

(15) $$\begin{array}{c} f=\left[\begin{array}{cc} F_{1} & F_{2}\\ E_{1} & E_{2}\\ A_{1} & A_{2} \end{array}\right] \end{array}$$

### 3.4. Twin Support Vector Machine

TWSVM, which is developed on the basis of traditional SVM, is a new machine learning method [23]. For the two-classification problem, TWSVM constructs a hyperplane for every class of samples, so that each class sample is closest to its own hyperplane and far away from another hyperplane. TWSVM solves two-classification problem by solving a set of quadratic programming problems (QPPs), and SVM solves all classification problems by solving one QPP. This strategy makes TWSVM work 4 times faster than a standard SVM [24].

Combine OVO strategy with standard TWSVM to get OVO TWSVM, and the OVO TWSVM has a better classification performance than the OVO SVM [25]. For *k* class classification problem, the algorithm constructs a two-classification TWSVM subclassifier between any two classes of samples. Each subclassifier in OVO TWSVM needs only two classes of samples for training. Two hyperplanes are needed to train two types of sample *i* and *j*, such as

(16) xTwij+bij=0

(17) xTwji+bij=0

where *w*_*ij*_ and *w*_*ji*_ are two normal vectors of hyperplanes and *b*_*ij*_ and *b*_*ji*_ are two hyperplanes. It is generally obtained by solving the following two-quadratic programming problem:

(18) min 12Aiwij+eij1bij2+cij2eij2Tξijs.t. Aiwij+eij2bij+ξij≥eij2,ξij≥0

(19) min 12Ajwji+eij2bji2+cji2eij2Tξjis.t. Ajwji+eij1bji+ξji≥eij2,ξji≥0

where *c*_*ij*_ is a penalty parameter and *e*_*ij*_ is a column vector of 1.

For the nonlinear separable phenomenon of training data, OVO TWSVM needs to solve the following optimization problems when training two samples of *i* and *j*:

(20) min 12KAi,CTwji+eij1bji2+cij2eijTξijs.t. KAi,CTwji+eij2bji+ξji=eij2,ξij≥0

(21) min 12KAi,CTwji+eij2bji2+cji2eijTξjis.t. KAi,CTwji+eij1bji+ξji=eij1,ξji≥0

**Figure 6:**
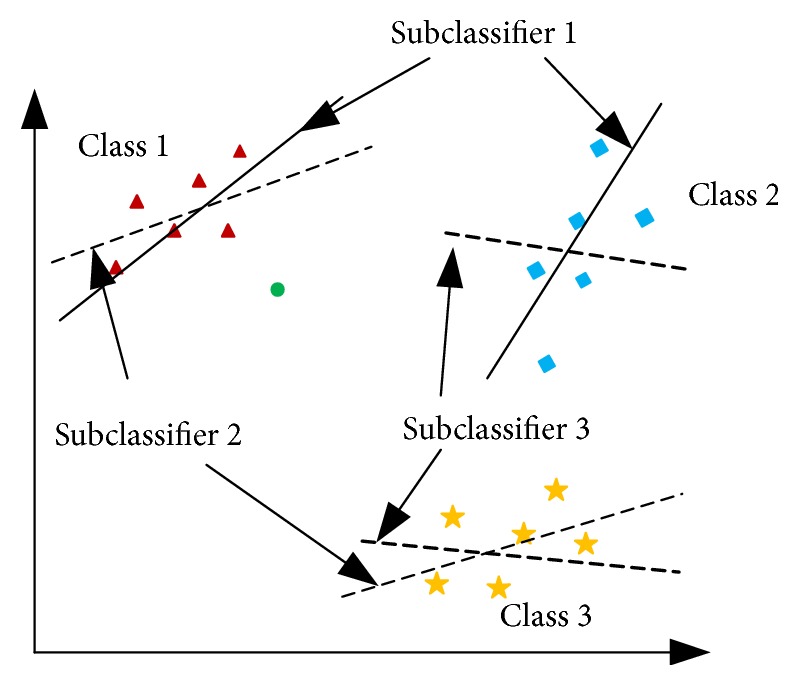
The sketch map of OVO TWSVM.

Taking three-classification problem in two-dimensional space as an example to illustrate the process of OVO TWSVM with Figure 6. Taking *i* and *j* in (21) and (22) as 1 and 2 and solving them, we can get a two-class OVO TWSVM that classifies class 1 and class 2, i.e., subclassifier 1 in Figure 6. The subclassifier 2 and the subclassifier 3 can be obtained by a similar method, which constitute the OVO TWSVM for solving the three-classification problem together. Taking the green dot in the figure as an example to illustrate how to use OVO TWSVM. First, calculate the distance from the dot to the two hyperplanes of subclassifier 1; because the dot is close to the hyperplane of class 1, class 1 gets 1 vote. Second, calculate the distance from the dot to the subclassifier 2 in the same manner; since the dot is close to one hyperplane of subclassifier 2 in class 1, class 1 gets 1 more vote. Third, calculate the distance from the dot to the subclassifier 3; because the dot is close to one hyperplane of subclassifier 3 in class 2, class 2 got 1 vote and class 3 got 0 vote. OVO TWSVM classify the classified sample into class 1.

For the three-classification problem of this article, three OVO TWSVM subclassifiers need to set 6 penalty parameters: *c*_11_, *c*_12_, *c*_21_, *c*_22_, *c*_31_, and *c*_32_.This article sets *c*_11_=*c*_21_=*c*_31_, *c*_12_=*c*_22_=*c*_32_.

### 3.5. Multiobjective Genetic Algorithm

The core of the multiobjective genetic algorithm is to coordinate the relationship between the target functions and to make the target functions reach the Pareto optimal set. The quality of a solution in the Pareto optimal set is defined according to the dominance criterion. Any solution *λ* of the Pareto optimal set can be seen as an acceptable solution. If a solution *λ*_1_ is no worse than *λ*_2_ in all objectives and *λ*_1_ is better than *λ*_2_ in at least one objective, then we define *λ*_1_ dominates *λ*_2_. The multiobjective optimization problem can be stated as

(22) Maxmize Fω=f1ω,f2ω,…,fnω

where *f*_1_(*ω*), *f*_2_(*ω*),…, *f*_*n*_(*ω*) are the *n* objective functions and *ω* represents the parameters of the model.

The NSGA algorithm based on the fitness sharing technique proposed by Goldberg is based on the principle of nondominated sorting to classify individuals in the population. And it can obtain a uniformly distributed Pareto optimal set or noninferior solution. However, the shortcomings of the algorithm are that the computational complexity is high and the sharing parameters need to be designated by human beings. Therefore, Deb's NSGA-II algorithm, which introduces fast nondominated sorting and elitist strategy to define crowding distance instead of fitness sharing, reduces the complexity of the algorithm and improves the computation efficiency. NSGA-II overcomes three shortcomings of NSGA: the computational complexity which dropped from* O*(*MN*^3^) to* O*(*MN*^2^) (where* M* is the number of objectives and* N* is the population size), an elitist-preserving approach, and no sharing parameters which need to be specified. More details can be seen at [26].

**Figure 7:**
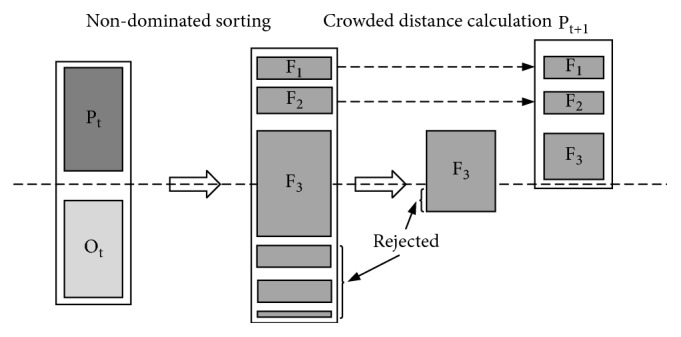
The steps of NSGA-II algorithm.

Figure 7 describes the application of the dominance criterion in the NSGA-II algorithm. NSGA-II algorithm starts from an initialization population *P*_*t*_ and each individual in a population is no worse than the remaining individuals in the population. The following steps combine the NSGA- II with OVO TWSVM to optimize the classification results of OVO TWSVM. And then generate offspring *O*_*t*_ from *P*_*t*_ through binary tournament selection, crossover, and mutation. Once a foreign source is obtained, the algorithm will combine the current population and the current generation into a group and classify them according to the nondominated sorting and crowding distance. *N* optimal solutions can be obtained in the final set.

## 4. Analysis of Experimental Results

### 4.1. Construction of the Objective Function

Using NSGA- II to optimize the parameters, this paper uses the correct rate to construct the target function. The objective function is given as follows:

(23) CRF=CNFTNF×100

(24) CRE=CNETNE×100

(25) CRA=CNATNA×100

(26) CR=CNF+CNE+CNATNF+TNE+TNA×100

*CRF*,* CRE*, and* CRA* represent the correct rate of flexion, extension and abduction,* CNF*,* CNE*, and* CNA* represent the correct action number of flexion, extension, and abduction, and* TNF*,* TNE*, and* TNA* represent the total number of flexion, extension, and abduction.* CR* represents the total correct rate.

### 4.2. Processing Steps

In the training phase, as the search process goes deep, the whole population tends to gather the global Pareto optimal set until the maximum evolutionary algebra is reached. The process of multiobjective optimization usually has the following steps:

(1) The dataset can be divided into a training set and a test set (50% for training, 50% for testing) or 5-fold cross validation, and in this article we use 5-folder cross validation in the whole analysis process.

(2) Change the parameters of the OVO TWSVM and run the target function.

(3) Set evolution algebra or stop criteria [27].

(4) Analyze the global optimal set to get the optimal parameters.

Table 1 shows the preset parameters of NSGA-II algorithm. The population size of the article is 100 and the crossover rate and mutation rate are 0.9 and 0.1.

Table 2 shows the range of penalty parameters and kernel function width for OVO TWSVM.

Table 3 shows the partial Pareto optimal set of the subject A and the corresponding OVO TWSVM model parameters using the NSGA-II algorithm.

**Figure 8:**
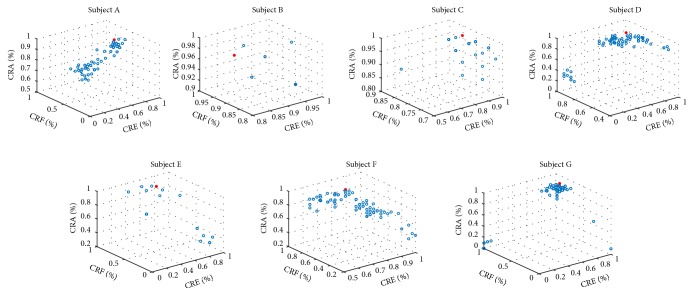
The Pareto optimal front of seven subjects based on RBF kernel OVO TWSVM.

Figure 8 shows the Pareto optimal fronts constructed by all nondominated solutions when seven subjects reach the optimal recognition rate. The mark points represent the optimum solution.

**Figure 9:**
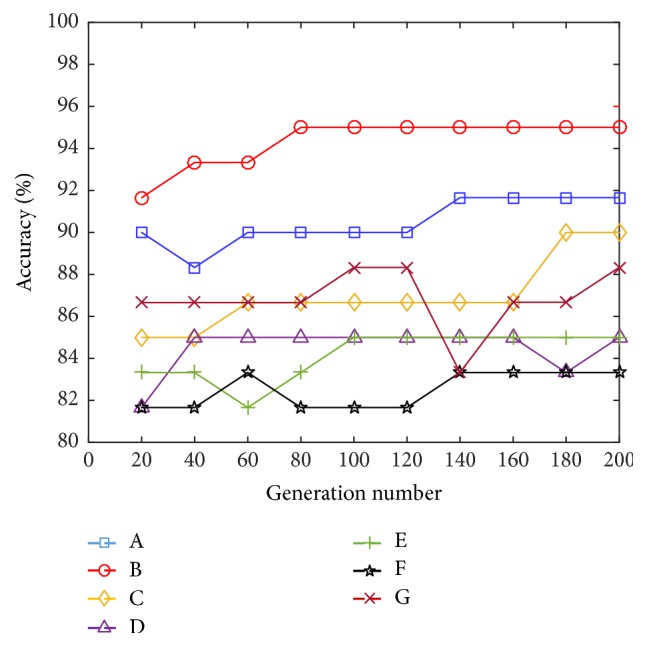
Evolutionary convergent fold line chart for different subjects.

Figure 9 shows the evolutionary convergence curve of different subjects. It can be seen from the figure that five of the seven subjects achieved the highest recognition rate within 200 generations, they are subjects A, B, C, E, and F, and their accuracies are 91.66%, 95.00%, 90.00%, 85.00%, and 85.00%. All the subjects continued to increase the evolution algebra until the 600 generations. It was found that two subjects D and G had converged in the 200 generation, namely, 85% and 88.33%, respectively. The recognition rate of subjects F increased to 85% in the 400 generations and remained stable within 600 generations.

**Figure 10:**
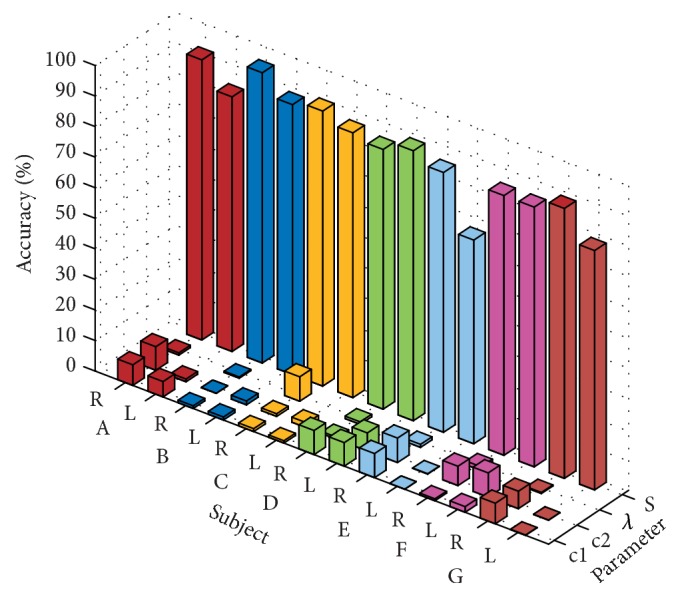
OVO TWSVM recognition effect diagram of different kernel function types.

Figure 10 shows the accuracy of the OVO TWSVM classification using the linear kernel and the RBF kernel, respectively, along with the corresponding penalty parameters* c1*,* c2 *and kernel function width *λ*, and S represent different subject. As can be seen from Figure 10, OVO TWSVM based on RBF kernel achieved the highest recognition rate on six subjects, up to 95% on subject B. Subject D's highest recognition rate appeared in linear kernel based OVO TWSVM, but the recognition result was only 3.33% less than that of RBF kernel based OVO TWSVM. Therefore, this paper chooses RBF kernel based OVO TWSVM as the final recognition model.

### 4.3. Comparison with Other Methods

**Figure 11:**
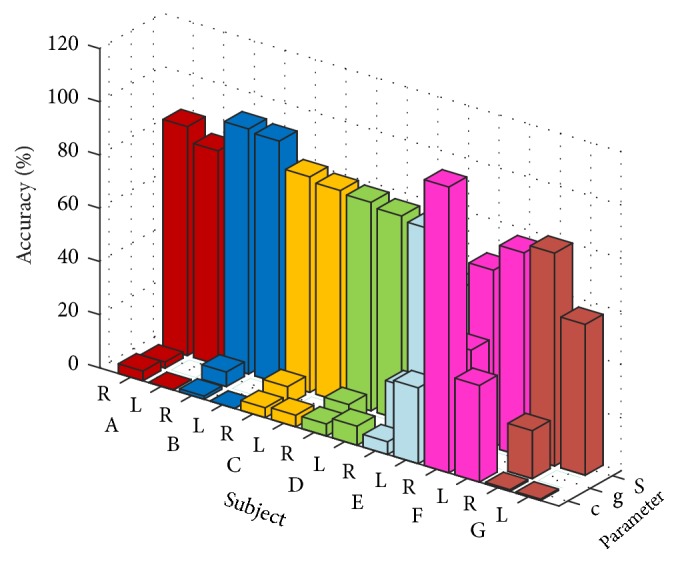
LS-SVM recognition rate diagram for different kernel functions.

Literature [20] uses LS-SVM to classify the motor imagery EEG signals; this paper applies the model to our own dataset for classification. Fivefold cross validation for each person's data, the results, and the corresponding parameter settings are shown in Figure 11. Where *c* is the penalty parameter, *g* is the kernel function width. As can be seen from Figure 11, LS-SVM with RBF kernel has the highest recognition rate of 92.09% on subject B, five other subjects were over 75%, and the worst recognition rate on subject F is 66.66%. Based on the above results, it can be considered that this method performs well on the data sets of different subjects.

**Figure 12:**
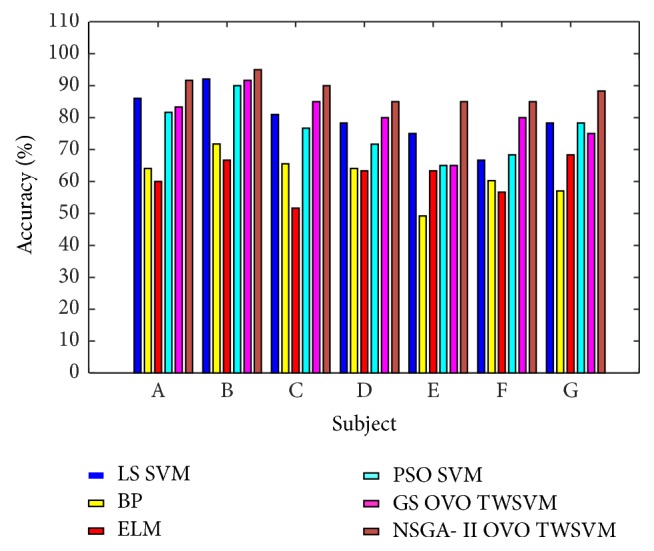
Comparison of recognition rates of different methods.

We also use BP with momentum, ELM, PSO-SVM, and grid search OVO TWSVM to classify the datasets. The result of the classification is shown in Figure 12. It can be seen from Figure 12 that the proposed method (88.57%±3.61%) has the highest average recognition rate among the seven subjects compared to other recognition algorithms. LS-SVM (79.64%±7.47%), GS OVO TWSVM (79.99%±7.76%), and PSO-SVM (75.95%±7.86%) have similar average recognition effect on seven subjects, and the average classification results of BP (61.70%±6.62%) and ELM (61.42%±5.37%) in this dataset are lower than that of SVM classifier. This shows that SVM classifier has a great advantage in small sample machine learning.

Since the time domain signal is more similar, especially in the single joint motor imagery EEG signal, in this paper, we combine amplitude-frequency (AF) domain information with CSP, namely, AF-CSP to get feature vectors. Different from the traditional method of combining time domain signal with CSP to construct feature vector, AF information can reveal further the difference between different actions in frequency domain and amplitude range; thus, CSP can get stronger feature extraction ability. In this paper, we compare the classification rate using AF-CSP and CSP directly after EMD, as shown in Table 4. As can be seen from Table 3, the proposed method has achieved a higher recognition rate among the 5 subjects, and it might be interpreted that AF-CSP is a more effective feature extraction method.

In order to distinguish the significance of the proposed method in this article, the one-way analysis of variance (ANOVA) method was used to compare with the other five algorithms. The* p*-value values are shown in Table 5, where* p1*,* p2*,* p3*,* p4*, and* p5* represent the p-values between the proposed method and LS-SVM, BP, ELM, PSO-SVM, and grid search OVO TWSVM. When* p*≤0.05, there is a significant difference in the recognition effect between the two algorithms. It can be seen from Table 5 that the* p*-values between the proposed method and the five-other methods are significantly less than 0.01, which proves that the proposed algorithm has significant classification in improvement performance compared with other algorithms.

## 5. Discussion

Common spatial pattern is widely used in motor imagery to extract EEG features [28]. This method uses supervised learning to obtain two types of filter to separate two motor imagery tasks. In recent years, several methods like CSSP [29], RCSP [30], SSCSP [31], FERCSP [32], SBCSP [33], and FBCSP [34] have been used to improve conventional CSP. But the drawback of CSP is that it needs a lot of electrodes. AF-CSP, which taking into account the AF information in the EEG signals, uses only four electrode channels to achieve a better recognition effect than conventional CSP.

And an optimized OVO TWSVM using NSGA-II is used to improve the accuracy of motor imagery; the mean accuracy of the proposed method is 88.57%±3.61%. However, since there are no articles consistent with the contents of this paper, we can only discuss articles that are similar to our research contents. Literature [18] points out that the manual extraction of features in the traditional biological signal pattern recognition model may produce information loss; thus, CNN with deep learning is introduced to identify of changes in the brain topographic map. The results show that three kinds of motor imagery in the hand are 65.51% and the kappa value in this experiment is 0.481. However, there is still a great deal of uncertainty about the intention recognition of single hand. In [19], the wavelet coefficients are calculated from EEG signals as feature and employed including quadratic discriminant analysis, naive Bayes quadratic, decision tree, K nearest neighbors, and SVM classifiers to identify shoulder and elbow joint movement. The highest recognition rate is 84.91%, using the SVM classifier. The weakness of this study is that it only recognizes the shoulder and elbow joint movements of one hand and there is no further analysis of the complex movement of the single joint.

It should be noted that we use AF-CSP to extract features and enhance the feature extraction ability of CSP. OVO TWSVM is used to identify three types of upper limb movement; the lowest recognition rate is 85.00%. Compared with the current research, we have made a more in-depth analysis of single joint multiclass motor imagery. A more sophisticated analysis can be done using the mean confusion matrices given in Table 6 and the kappa value calculated by Table 6 is 0.82. It appears that the extension action is more difficult to distinguish with flexion and abduction of the shoulder joint using the proposed method. The flexion and extension of the shoulder joint obtain high recognition rate by the proposed method.

In addition, [35] proposes a novel correlation-based time window selection (CTWS) algorithm which considers the variation in the time latency during the MI task for MI-based BCI. CTWS adjusts the starting points of time window for both training and test samples using correlation analysis and shows significantly improvement than feature extraction algorithms without CTWS. As the feature extraction algorithms in the structure of the CTWS algorithm is substitutable, the focus of the next work is to combine CTWS with AF-CSP to increase the recognition rate of the extension and applying this model to the BCI systems.

## 6. Conclusion

This paper proposes AF-CSP replace the traditional EMD-CSP method. The main idea of this method is to analyze the *μ* and *β* rhythm information contained in each IMF component after EMD decomposition and extract corresponding AF information. This preprocessing method not only removes the influence of irrelevant frequency bands, but also strengthens the feature extraction ability of CSP. Secondly, this paper also uses NSGA-II to optimize the OVO TWSVM parameter optimization process. Compared with other evolutionary strategies, the Pareto optimal set obtained by NSGA-II can make OVO TWSVM more robust. In the future, we may use this technology to evaluate the classification of real-time BCI and apply this method for reach and grasp tasks of a robotic arm. In order to improve the recognition rate of the method, we may use clustering or dimensionality reduction method to get a more obvious feature vector.

## Figures and Tables

**Table 1:** NSGA- II preset parameters.

**Parameter**	**Name**	**Preset value**
P	Population size	100
CR	Crossover rate	0.9
MR	Mutation rate	0.1

**Table 2:** OVO TWSVM parameter range.

**Parameter**	**Name**	**Lower limit**	**Upper limit**
*c1*	Penalty parameter 1	2^−3^	2^3^
*c2*	Penalty parameter 2	2^−3^	2^3^
*λ*	RBF kernel function	2^−20^	2^3^

**Table 3:** Pareto optimal set of subject A and the corresponding model parameters.

***c1***	***c2***	***λ***	***CRF***	***CRE***	***CRA***	***CR***
0.2031	0.0100	0.7810	0.95	0.85	0.90	0.9000
5.0102	5.7356	0.1130	0.40	0.20	1.00	0.5333
5.4325	6.6230	0.2384	0.75	1.00	0.65	0.8000
**6.7280**	**7.7008**	**0.7514**	**0.95**	**0.95**	**0.85**	**0.9166**
4.1136	0.0100	0.6551	0.95	0.85	0.90	0.9000
4.8389	7.3435	0.2332	0.80	1.00	0.60	0.8000
3.6172	4.8072	0.1111	0.45	0.15	1.00	0.5333

**Table 4:** Comparison between AF-CSP and EMD-CSP.

**Subjects**	**A**	**B**	**C**	**D**	**E**	**F**	**G**	**Mean**
AF-CSP	**91.66**	**95.00**	**90.00**	**85.00**	85.00	85.00	**86.67**	**88.57**%**±3.61**%
EMD-CSP	88.33	88.33	81.66	76.67	**88.33**	**83.34**	73.34	82.85%±5.61%

**Table 5:** *p*-value score based on RBF kernel OVO TWSVM and five other methods.

**Subject**	***p1***	***p2***	***p3***	***p4***	***p5***
A	1.06e-05	3.90e-07	6.29e-08	2.78e-08	7.22e-05
B	3.80e-02	9.72e-06	6.97e-13	1.00e-03	5.33e-04
C	1.07e-07	6.27e-09	3.60e-11	3.55e-05	2.37e-07
D	2.84e-05	3.99e-07	9.81e-07	6.09e-07	3.00e-04
E	1.00e-04	3.64e-12	3.04e-06	1.84e-06	2.61e-11
F	3.88e-07	8.30e-08	9.84e-08	5.93e-06	3.40e-03
G	3.00e-04	3.82e-09	7.56e-06	4.91e-05	5.94e-07

**Table 6:** Mean confusion matrices of seven subjects for the proposed method.

	**Flexion**	**Extension**	**Abduction**
**Flexion**	90.71	7.57	3.50
**Extension**	5.50	82.14	5.79
**Abduction**	3.93	10.26	90.71

## Data Availability

The data used to support the findings of this study are available from the corresponding author upon request.
